# MicroRNA-20a-5p Ameliorates Non-alcoholic Fatty Liver Disease via Inhibiting the Expression of CD36

**DOI:** 10.3389/fcell.2020.596329

**Published:** 2020-12-03

**Authors:** Xin Wang, Yan Ma, Long-Yan Yang, Dong Zhao

**Affiliations:** ^1^Center for Endocrine Metabolism and Immune Diseases, Beijing Luhe Hospital, Capital Medical University, Beijing, China; ^2^Beijing Key Laboratory of Diabetes Research and Care, Beijing, China

**Keywords:** fatty acid translocase CD36, non-alcoholic fatty liver disease, microRNA-20a-5p, lipid, deposition

## Abstract

Fatty acid translocase CD36 (CD36) plays an important role in the initiation and pathogenesis of chronic liver disease and non-alcoholic fatty liver disease (NAFLD). The purpose of this study is to investigate the regulation of microRNA-20a-5p (miR-20a-5p) on CD36 in the pathogenesis of NAFLD. Human plasma samples were obtained from NAFLD patients and healthy controls. Mice were fed with high-fat diet to induce an *in vivo* NAFLD model. Histology staining was performed to examine the morphology and lipid deposition of mouse liver tissue. Real-time PCR, dual-luciferase assay, and western blotting were employed to detect the relationship between miR-20a-5p and CD36. The expression level of miR-20a-5p was decreased in NAFLD patients, HFD mice, and free fatty acid (FFA)-treated HepG2 cells or primary mouse hepatocytes, accompanied by increased lipid production in hepatocytes. MiR-20a-5p suppressed the expression of CD36 to reduce lipid accumulation via binding to its 3’-untranslated region (UTR). However, under the condition of interference with CD36, further inhibition of miR-20a-5p would not cause lipid over-accumulation. In this study, we found that miR-20a-5p played a protective role in lipid metabolic disorders of NAFLD by targeting CD36, which indicated the prospect of miR-20a-5p as a biomarker and treatment target for NAFLD.

## Introduction

Non-alcoholic fatty liver disease (NAFLD) is now considered as the most common chronic liver disease worldwide. The prevalence of NAFLD has been on the rise from 15% in 2005 to 25% in 2010, and its advantage stage non-alcoholic steatohepatitis (NASH) has almost doubled a similar rate in the same timeframe (33–59.1%) (Younossi et al., 2016, 2018). Due to the increasing impact on world health, there have been extensive related studies in NAFLD and its histological phenotype NASH, which is characterized by steatosis, inflammation, ballooning, and fibrosis (Schwimmer et al., 2005). Growing evidence suggests that NASH can subsequently progress to liver fibrosis, cirrhosis, and hepatocellular carcinoma (HCC) (Younossi et al., 2016). However, the pathogenesis of NAFLD is complex, and there is still lack of a peculiar treatment that can block the occurrence and progression.

As a membrane of glycoprotein, fatty acid translocation enzyme CD36 (CD36) is weakly expressed in hepatocytes and liver tissue under physiological conditions but significantly increased in animal models and NAFLD patients (Greco et al., 2008; Garcia-Monzon et al., 2014), while overexpression of CD36 led to steatosis in mice, and liver-specific knockout of CD36 reduced lipid content in mice fed with high fat diet (HFD) (Koonen et al., 2007; Wilson et al., 2016). It has been clearly confirmed that CD36 can affect FA oxidation by affecting the activation of AMPK (Samovski et al., 2015), while it enhances insulin signaling (Wallin et al., 2010). More importantly, CD36 is also involved in regulating chronic metabolic inflammation by activating the JNK pathway (Kennedy et al., 2011). A recent study suggested that osteoprotegerin (OPG) can regulate CD36 transcription through the ERK-PPARγ pathway, thus promoting hepatic steatosis (Zhang et al., 2019a).

MicroRNAs (miRNAs) are short small non-coding RNA molecules, which can directly cause mRNA degradation or translation inhibition by pairing with the 3′ untranslated region (UTRs) of the mRNA (Bartel, 2004). Many studies have demonstrated that serum/plasma miRNAs can be used as biomarkers for NAFLD. In addition, an early study has shown that four kinds of serum miRNAs, miR-21, miR-34a, miR-122, and miR-451, which are involved in regulating the lipid accumulation and metabolism, were significantly higher in the liver of NAFLD patients than in those with non-NAFLD (Yamada et al., 2013). In Fang et al.’s (2016) study, miR-20a-5p, as a member of the miR-17 family, may promote liver glycogen synthesis and be involved in hepatic insulin signaling induced by high glucose by targeting p63 to regulate the expression of p53 and PTEN. However, the direct effect and underlying mechanisms of miR-20a-5p in NAFLD and lipid deposition are still unclear. Therefore, in the present study, we investigated the effect of miR-20a-5p in NAFLD and explored the potential mechanisms. The study will provide fundamental information as to its suitability as a target for the treatment of NAFLD.

## Materials and Methods

### The Clinical Data and Patient Samples

The clinical data and plasma samples of patients were collected from Beijing Luhe Hospital, Capital Medical University (Beijing, China), which included healthy controls (*n* = 13) and NAFLD patients (*n* = 14). All participants were aged 30–48. NAFLD was diagnosed by using ultrasound based on the statement of the American Gastroenterological Association in 2012 (Lee et al., 2010; Chalasani et al., 2012). The information on liver stiffness measurement (LSM) was detected by transient elastography (TE), a non-invasive technique, to evaluate the degree of fibrosis of these patients (Castera et al., 2008). Individuals with any other systemic inflammatory diseases, viral hepatitis, alcoholic hepatitis, or autoimmune hepatitis were excluded. The entire study was obtained ethical approval from the competent Institutional Review Boards of Beijing Luhe Hospital, Capital Medical University.

### Animal Model

Male C57BL/6J mice at the age of 8 weeks were purchased from Charles River (Beijing, China) and fed with standard chow diet or high-fat diet (HFD) for 16 weeks. All mice received humane treatment and were maintained in specific pathogen-free conditions. All animal protocols were approved in accordance with the guideline of Ethics Committee of the Capital Medical University, Beijing, China.

### Cell Culture

HepG2 cells and HEK-293T cells were cultured with Dulbecco’s Modified Eagle’s Medium (DMEM) supplemented with 10% fetal bovine serum (FBS) and 1% penicillin/streptomycin (P/S) at 37°C with 5% CO_2_ in an incubator. Primary mouse hepatocytes were isolated from the liver of 8-week male C57BL/6J mice (Denizeau et al., 1985). Hepatocytes were cultured with RPMI 1640 (Gibco, United States) supplemented with 10% FBS and 1% P/S at 37°C with 5% CO_2_ in an incubator. To induce steatosis, 5 × 10^5^ cells were plated into six-well plates for 24 h and then stimulated with 1 mM FFA mixture [oleate (OA) and palmitate (PA) at a final ratio of 2:1] in DMEM for 48 h (Ye et al., 2018a). OA and PA were dissolved in 10% fatty acid-free bovine serum (BSA). Therefore, 10% of BSA was used as a control for FFA stimulation.

### Transfection of SiRNA and MiRNA

To implore the effect of miR-20a-5p, we transfected miR-20a-5p mimic, inhibitor, or miR-negative control (Ribobio, Guangzhou, China) into HepG2 and HEK-293T cells by using Lipofectamine^TM^ 2000 (Thermo Fisher Scientific, United States). The following siRNAs against CD36 (siCD36) were used: human siCD36-1 sense (5′–3′), CCCUGUUACUACCACAGUUdTdT; antisense (5′–3′), AACUGUGGUAGUAACAGGGdT dA; human siCD36-2 sense (5′–3′), CCCUGUGUAUAGAUUUGUUdTdT; antisense (5′–3′), AACAAAUCUAUACACAGGGdAdT; human siCD36-3 sense (5′–3′), CCUAUAACUGGA UUCACUUdTdT; antisense (5′–3′), AAGUGAAUCCAGUUAUAGGdTdT.

### Measurement of Lipids in Serum, Liver Tissues, and Cells

After 16 weeks of HFD, mice were sacrificed at the end of the experiments; serum was collected for the assay of alanine aminotransferase (ALT), aspartate aminotransferase (AST), triglyceride (TG), and cholesterol (TC) with the Hitachi 7600-020 clinical analyzer (Hitachi, Tokyo, Japan).

For HepG2 cells and liver tissues, the cellular lipid content of TG was measured by commercial kits (Jiancheng Bioengineering Institute, Nanjing, China).

### Western Blotting

Liver tissue samples and HepG2 cells were lysed with RIPA lysis buffer (Apply Gene, Beijing, China) containing 1% PMSF. The primary antibodies against CD36 (1:1,000, Abcam, Cambridge, United Kingdom), GAPDH (1:5,000, Proteintech, Chicago, United States), FATP2 (1:1,000, Proteintech, Chicago, United States), and FATP5 (Bioss, Beijing, China) were used. After incubating overnight at 4°C, the corresponding HRP-conjugated anti-rabbit IgG secondary antibodies (1:5,000, Proteintech, Chicago, IL, United States) were used.

### Real-Time PCR (RT-PCR)

Total RNA from human plasma was isolated by using the TRIzol LS (Invitrogen, United States), while total RNA from mouse liver and HepG2 cells were extracted with TRIzol (Invitrogen, United States) according to the manufacturer’s instructions. To get cDNA sequence and quantify the expression of miR-20a-5p, total RNA was reversed by using a commercial MicroRNA Reverse Transcription Kit (Ribobio, Guangzhou, China). To get cDNA sequence and quantify the expression of mRNA, the Reverse Transcription Kit and SYBR Green Supermix (Bio-Rad, CA, United States) were used. The following PCR primers were used: human CD36 forward, 5′-GGCTGTGACCGGAACTGTG-3′; reverse, 5′-AGGTCTCCAACTGGCATTAGAA-3′; human FATP2 forward, 5′-TACTCTTGCCTTGCGGACTAA-3′; reverse, 5′-CCGAAGCAGTTCACCGATATAC-3′; human FATP5 for- ward, 5′-GCTTCGGTCCTATTCGGATCT-3′; reverse, 5′-CAG CGCCCCACATAGTTGA-3′; mouse CD36 forward, 5′-AAG CCAGGTATTGCAGTTCTTT-3′; reverse, 5′-GCATTTGCTGA TGTCTAGCACA-3′; mouse FATP2 forward, 5′-TACTCTTGC CTTGCGGACTAA-3′; reverse, 5′-CCGAAGCAGTTCACCGA TATAC-3′; mouse FATP5 forward, 5′-GCTTCGGTCCTATT CGGATCT-3′; reverse, 5′-CAGCGCCCCACATAGTTGA-3′; 18s, forward, 5′-GTAACCCGTTGAACCCCATT-3′; reverse, 5′-CCATCCAATCGGTAGTAGCG-3′.

### Histology Staining

Five-micrometer-thick liver tissue slices were stained with hematoxylin and eosin (H&E), Oil-Red O, and Masson (Servicebio, Wuhan, China). The hepatic histology was observed and captured by light microscopy DM500 B (Leica Microsystems, Wetzlar, Germany).

### Dual-Luciferase Assay

The wild-type (WT-CD36) and mutant (MUT-CD36) three prime untranslated regions (3′ UTRs) of CD36 mRNA were cloned and inserted downstream of the GV657 vector (Genechem, Shanghai, China), then 1 μg WT-CD36 and MUT-CD36 plasmid were transfected into HepG2 and HEK-293T cells with miR-20a-5p mimics or inhibitors for 48 h by using Lipofectamine 2000. For luciferase activity measurement, the Dual-Luciferase Assay Kit (Promega, United States) was used by following the manufacturer’s instructions. The results of luciferase activity were presented as the ratio of Renilla luciferase activity to firefly luciferase activity.

### Statistical Analysis

Experimental results are presented as means ± SEM. Comparisons between the two groups were performed with GraphPad Prism 7 (San Diego, CA, United States) software. Student’s *t*-test or ANOVA was used to determine the statistical significance between two or more than two groups. *P* < 0.05 was considered statistically significant.

## Results

### The Expression Level of MiR-20a-5p Is Decreased in NAFLD Patients and Mice

**FIGURE 1:**
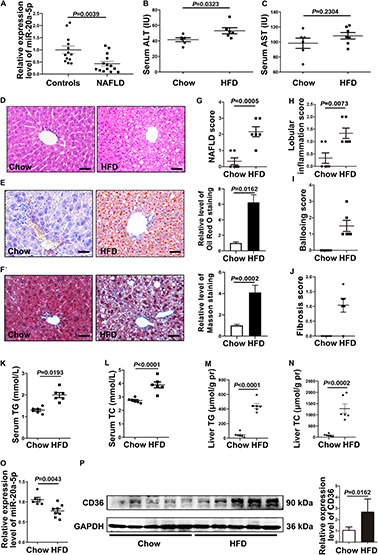
The expression level of miR-20a-5p is decreased in NAFLD patients and HFD mice. **(A)** Real-time PCR analysis of miR-20a-5p expression levels in the plasma of healthy controls (*n* = 13) and NAFLD patients (*n* = 14). **(B,C)** Mouse serum, ALT, and AST levels were measured to evaluate liver function (*n* = 6 mice per group). **(D)** Hematoxylin and eosin staining of mouse liver tissue (*n* = 6 mice per group). Scale bars: 50 μm. **(E)** Oil red O staining revealed the reality of lipid deposition in mouse liver tissue; the results quantified three images/mouse by Image-Pro Plus (*n* = 6 mice per group). Scale bars: 50 μm. **(F)** Masson staining revealed the reality of liver fibrosis in mouse liver tissue; the results quantified three images/mouse by Image-Pro Plus (*n* = 6 mice per group). Scale bars: 50 mm. **(G–J)** Mouse hepatic NAFLD, lobular inflammation, ballooning, and fibrosis score. **(K–N)** TG and TC contents in serum and liver tissue of mice were measured (*n* = 6 per group). **(O)** RT-PCR analysis of miR-20a-5p expression levels in the liver of mice after HFD (*n* = 6 per group). **(P)** Western blot analysis of CD36 protein levels in mouse liver tissues. Data were mean ± SEM. NAFLD, non-alcoholic fatty liver disease; HFD, high-fat diet; RT-PCR: real-time polymerase chain reaction; ALT, alanine aminotransferase; AST, aspartate aminotransferase; TG, total triglyceride; TC, total cholesterol.

Aiming to decipher whether the level of miR-20a-5p might be altered in NAFLD patients, we first carried out RT-PCR analysis with all 27 plasma samples, including 13 non-NAFLD subjects and 14 NAFLD patients. As shown in Figure 1A, the level of miR-20a-5p exhibited a significant decrease in the plasma of NAFLD patients compared with non-NAFLD subjects. This result implied that miR-20a-5p may play a role in the pathological process of NAFLD. The demographics and clinical characteristics of healthy controls and patients with NAFLD are shown in Table 1. The distribution of age and gender was similar in both groups. BMI, TG, and TC levels were significantly higher in NAFLD patients. The NAFLD stage was defined by ultrasound into three grades: mild, moderate, and severe; almost 57% of patients were defined as mild; 29% of patients were defined as moderate; and 14% of patients were defined as severe. TE measurement showed that the LSM value of most patients was above the normal range (medium value = 6.6 kPa normal range: 2.6–6.2 kPa), which suggested that most of the patients may develop moderate fibrosis.

**TABLE 1:** Demographics and clinical characteristics of healthy controls and patients with NAFLD.

**Characters**	**Healthy controls (*n* = 13)**	**NAFLD (*n* = 14)**	***P*-value**
Age, years	36.2 ± 4.51	38.3 ± 5.18	0.2838
Gender, M/F	10/3	11/3	–
BMI (kg/m^2^)	24.0 ± 2.84	28.6 ± 2.89	0.0004
Smoking	No	No	–
Diabetes mellitus	No	No	–
Metabolic syndrome	No	No	–
Hypertension	No	No	–
Systolic blood pressure (mmHg)	120.1 ± 8.46	125.6 ± 20.4	0.7826
Diastolic blood pressure (mmHg)	71.7 ± 8.48	74.2 ± 14.7	0.593
AST (U/L)	18.9 ± 5.44	22.4 ± 11.4	0.3236
ALT (U/L)	24.3 ± 14.8	36.6 ± 27.5	0.1634
Total cholesterol, mmol/L (CHO)	1.31 ± 0.77	2.64 ± 1.26	0.003
Total triglyceride (TG)	4.27 ± 0.92	5.23 ± 0.68	0.0046
HDL cholesterol, mmol/L (HDL)	1.26 ± 0.19	1.23 ± 0.36	0.8108
LDL cholesterol, mmol/L (LDL)	2.51 ± 0.71	3.08 ± 0.91	0.0937
**NAFLD stage**			
Mild	–	8	–
Moderate	–	4	–
Severe	–	2	–

*Data was presented as mean value ± SD.*

To address this possibility, we induced the mouse NAFLD model by HFD; the serum ALT level was increased in the NAFLD group (Figure 1B), and the AST level remained unaffected compared with the non-NAFLD group (Figure 1C). Then, we examined the histopathology in NAFLD and non-NAFLD mouse liver tissue sections as confirmed by H&E staining (Figure 1D), Oil Red O staining (Figure 1E), and Masson staining (Figure 1F). After 16 weeks of high-fat diet, C57BL/6J mice developed typical hepatic steatosis (Figures 1D,E,G), lobular inflammation (Figure 1H), hepatocellular ballooning (Figure 1I), and moderate fibrosis (Figures 1F,J). Hepatic NAFLD score, inflammation, and fibrosis were all based on the Kleiner et al. (2005) scoring system to compare with the human NAFLD liver phenotype.

The serum TG and TC levels were increased in NAFLD mice compared with non-NAFLD mice (Figures 1K,L), and the liver TG and TC levels were also increased in NAFLD mice compared with non-NAFLD mice (Figures 1M,N). Meanwhile, the hepatic miR-20a-5p level in NAFLD mice was statistically lower than that in non-NAFLD mice (Figure 1O). These data suggested that the level of miR-20a-5p was decreased in both NAFLD patient and NAFLD mouse model induced by HFD.

### Downregulation of MiR-20a-5p Is Accompanied by the Upregulation of CD36 and Increased Lipid Deposition

**FIGURE 2:**
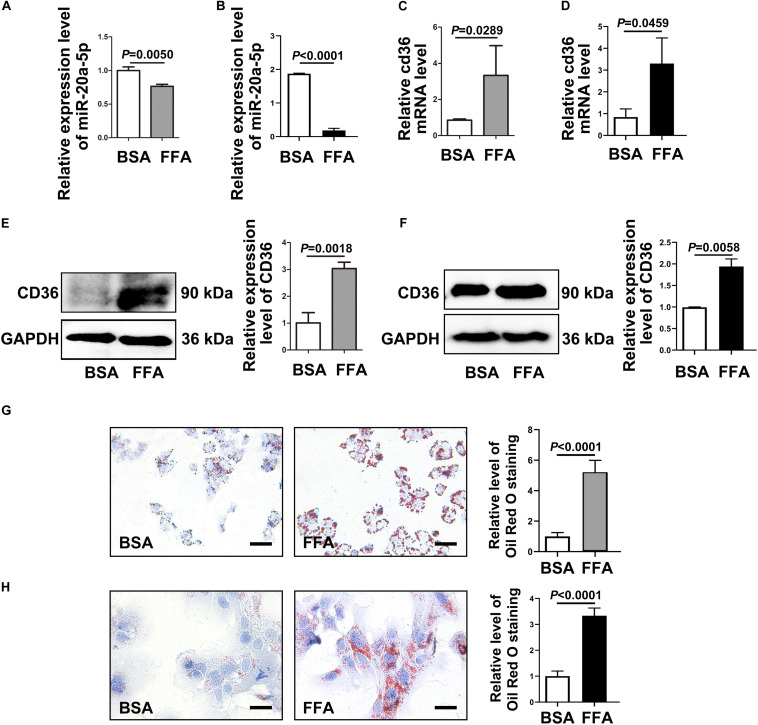
CD36 is inhibited by miR-20a-5p. After FFA-treatment, HepG2 cells **(A)** and primary mouse hepatocytes **(B)** were lysed to detect the miR-20a-5p expression levels. The mRNA levels of CD36 in HepG2 cells **(C)** and primary mouse hepatocytes **(D)** were measured after FFA-stimulation by RT-PCR. The protein levels of CD36 in HepG2 cells **(E)** and primary mouse hepatocytes **(F)** were measured after FFA-stimulation by western blot. The condition of lipid deposition in HepG2 cells **(G)** and primary mouse hepatocytes **(H)** was measured by oil red o staining; the relative level of oil red o staining was quantified at least five images at each group by Image-Pro Plus. Data were mean ± SEM for three independent experiments with at least three multiple holes in each group. Scale bars: 50 μm. FFA, free fatty acid; RT-PCR, real-time polymerase chain reaction; BSA, bovine serum albumin.

To investigate the mechanism of miR-20a-5p in the lipid accumulation pathological process, CD36 might be a downstream target gene of miR-20a-5p, which was found by software prediction. To assess this hypothesis, we first detected the expression level of CD36 in HFD and chow diet mice, as shown in Figure 1P; the expression of CD36 was significantly increased in HFD mice compared to control mice. HepG2 cells and primary mouse hepatocytes were treated with FFA to induce hepatocyte steatosis. After FFA treatment, the expression level of miR-20a-5p was downregulated in HepG2 cells in a time-dependent manner, which was consistent with the increased expression level of CD36 (Supplementary Figure 1). The expression levels of miR-20a-5p in FFA-treated HepG2 cells and primary mouse hepatocytes were reduced similar to HFD mouse liver and NAFLD patients (Figures 2A,B). Notably, the CD36 mRNA level was upregulated (Figures 2C,D) upon FFA treatment. In line with the mRNA level, western blot analysis also confirmed the upregulation of the CD36 protein level in FFA-treated cells, respectively (Figures 2E,F). Furthermore, oil-red O staining images showed increased lipid deposition following FFA-treatment (Figures 2G,H). Taken together, these data implied that the decreased miR-20a-5p in NAFLD may result in the increased CD36 expression levels.

### CD36 Acts as a Downstream Target of MiR-20a-5p

**FIGURE 3:**
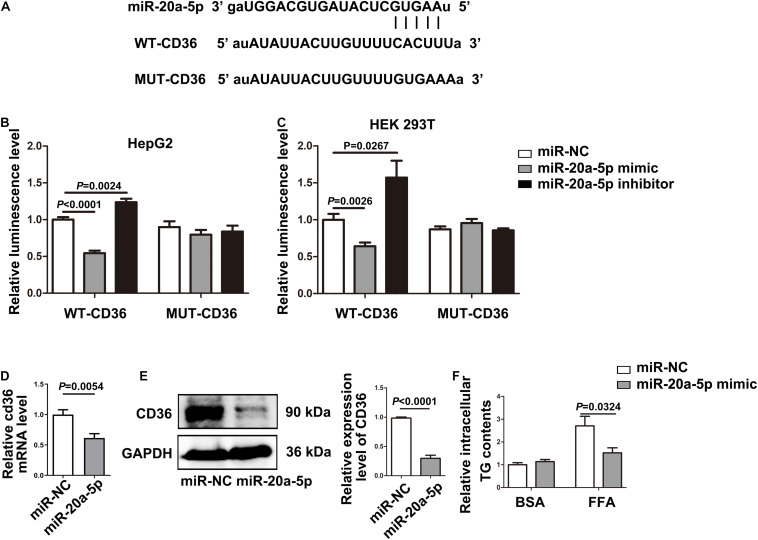
CD36 is identified as a downstream target of miR-20a-5p. **(A)** The binding site of miR-20A-5P on the 3′ UTR of CD36. **(B,C)** After transfecting the wild-type/mutant GV657-CD36 vector with the miR-20a-5p mimic/inhibitor in 293T and HepG2 cells, dual-luciferase reporter assay was performed to test the luciferase activity. **(D,E)** The mRNA and protein levels of CD36 in HepG2 cells were measured by RT-PCR and western blot after transfection of the miR-20a-5p mimic. **(F)** After transfection of the miR-20a-5p mimic, HepG2 cells were stimulated with FFA for 48 h, then the contents of total triglyceride in HepG2 cells were measured. Data were mean ± SEM. FFA, free fatty acid; WT, wild type; MUT, mutant; miR-NC, miRNA-negative control; BSA, bovine serum albumin; RT-PCR, real-time polymerase chain reaction.

The TargetScan analysis revealed that there is a binding site for miR-20a-5p on the 3′UTRs of CD36. To further test whether CD36 was a downstream target gene of miR-20a-5p, we initially cloned and amplified the wild-type or mutant 3′ UTR portion of CD36 into the GV657 vector (Figure 3A). After co-transfection of the wild-type 3′ UTR portion of the CD36 and miR-20a-5p mimic or miR-20a-5p inhibitor, overexpression of miR-20a-5p significantly reduced the luciferase activity, while downregulation of miR-20a-5p increased the luciferase activity in HepG2 (Figure 3B left). However, the luciferase activity was not obviously changed when HepG2 cells were co-transfected with the mutant 3′ UTR portion of the CD36 and miR-20a-5p mimic or miR-20a-5p inhibitor (Figure 3B right). It showed similar results in another cell line HEK-293T (Figure 3C). It is suggested that CD36 might be a target gene of miR-20a-5p. Moreover, overexpression of miR-20a-5p significantly suppressed the expression of CD36 in both mRNA (Figure 3D) and protein levels (Figure 3E) as well as TG contents in HepG2 cells (Figure 3F). In addition, downregulation of miR-20a-5p was shown to upregulate the CD36 protein level (Supplementary Figure 2). These data indicated that CD36 was a target gene of miR-20a-5p.

### MiR-20a-5p via Downregulation of CD36 to Reduce Lipid Accumulation

**FIGURE 4:**
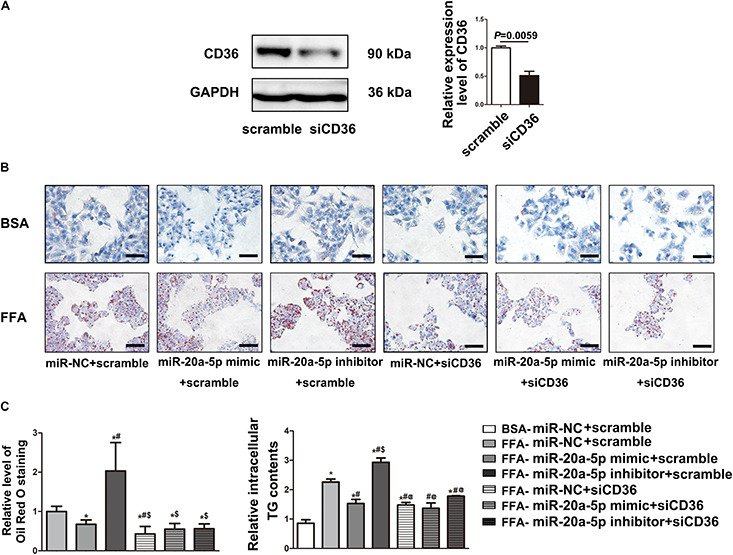
MiR-20a-5p via downregulation of CD36 to reduce lipid accumulation. **(A)** The protein level of CD36 in HepG2 cells was measured by western blot after transfection of siRNA-CD36. **(B)** After being transfected with miR-20a-5p mimic, inhibitor, or siRNA-CD36 alone or together, HepG2 cells were stimulated with FFA for 48 h, then the condition of lipid deposition in HepG2 cells was measured by oil red o staining. The relative level of oil red o staining was quantified at least five images at each group by Image-Pro Plus. Data were mean ± SEM for three independent experiments with at least three multiple holes in each group. *P < 0.05, different treatment groups vs. FFA-miR-NC+scramble; ^#^*P* < 0.05, vs. FFA-miR-20a-5p mimic+scramble; ^$^*P* < 0.05, vs. FFA-miR-20a-5p inhibitor+scramble. Scale bars: 100 μm. **(C)** After being transfected with miR-20a-5p mimic, inhibitor, or siRNA-CD36 alone or together, HepG2 cells were stimulated with FFA for 48 h. TG contents in HepG2 cells were measured by a commercial kit. Data were mean ± SEM. *P < 0.05, different treatment groups vs. BSA-miR-NC+scramble; ^#^*P* < 0.05, vs. FFA-miR-NC+scramble; ^$^*P* < 0.05, vs. FFA-miR-20a-5p mimic+scramble; ^@^*P* < 0.05, vs. FFA-miR-20a-5p inhibitor+scramble. BSA, bovine serum albumin; FFA, free fatty acid; miR-NC, miRNA-negative control; TG, total triglyceride.

To examine the miR-20a-5p-mediated regulation of CD36 in lipid degeneration, we transfected siCD36 into HepG2 cells; CD36 was efficiently knocked down to 50% (Figure 4A). After FFA treatment, Oil Red O staining analysis showed lower lipid accumulation in the miR-20a-5p and siCD36 transfection group, and even in the miR-20a-5p and siCD36 co-transfection group compared to control. In contrast, transfection with the miR-20a-5p inhibitor significantly aggravated lipid accumulation. Otherwise, co-transfection of siCD36 and the miR-20a-5p inhibitor could remarkably rescue the severe lipid accumulation phenomenon induced by the miR-20a-5p inhibitor alone (Figure 4B). Meanwhile, we also found similar results in the detection of cellular TG contents where the miR-20a-5p mimic and siCD36 significantly reduced the FFA-induced cellular TG level, whereas the miR-20a-5p inhibitor remarkably increased the cellular TG level and siCD36 transfection reduced the TG contents caused by the miR-20a-5p inhibitor (Figure 4C). Overall, these results suggested that miR-20a-5p could downregulate the CD36 protein level to alleviate lipid accumulation in hepatocytes.

## Discussion

The global incidence of NAFLD is as high as 25.2% (Younossi et al., 2016), from simple steatosis or non-alcoholic fatty liver disease, and more severe progressive diseases such as NASH were all included. However, there are few approved drugs or effective treatments for NAFLD. A better understanding of the pathogenesis of NAFLD is the only way to restrain the development of NAFLD. MiR-20a-5p is a member of the miR-17 family, which consists of six individual miRNAs (miR-17, miR-18a, miR-19a, miR-20a, miR-92a); all members of the miR-17 family share a common seed sequence (AAAGUG) (Mogilyansky and Rigoutsos, 2013). Early studies have shown that miR-17 family members are first identified in malignant B-cell lymphoma (Ota et al., 2004). Recent studies have demonstrated that the miR-17-92 cluster plays a critical role in the development of the immune system, heart, stroke, and various carcinogenic events (Xin et al., 2017; Zhang et al., 2018; Chen et al., 2019; Kuo et al., 2019). Several studies have confirmed the downregulation of miR-20a-5p in NAFLD patients and mouse models, suggesting that miR-20a-5p may play an important role in NAFLD and lipid deposition in the liver. In Ye et al.’s (2018b) study, by comparing the circulating miRNA expression profiles of type 2 diabetes mellitus (T2DM) patients with or without NAFLD, they found that the expression levels of plasma miR-17, miR-20a, miR-20b, and miR-122 are upregulated in T2DM patients with NAFLD but downregulated in the hepatic of T2DM complicated with NAFLD rat model. They also provided evidence that upregulation of the miR-17 family can target Phnox1 to rescue the insulin sensitivity and lipid metabolism (Ye et al., 2018a). MiR-20a-5p was also significantly reduced in the serum and liver specimens of NAFLD patients (Fang et al., 2016; Ando et al., 2019). In our study, the expression level of miR-20a-5p was significantly decreased in plasma samples of 14 human NAFLD patients and HFD-induced NAFLD mice liver tissues. Our results were consistent with the previous studies.

It has been reported that miR-20a-5p acted as a central hub that targeted about 500 genes, many of which play roles in signal transduction and T cell activation, and some of which have transcription factor activity (Angerstein et al., 2012). MiR-20a-5p was involved in many kinds of tumors, such as breast tumors and lung cancer (Zhao et al., 2018b; Guo et al., 2019; Zhang et al., 2019b). Moreover, a recent study showed that the low-density lipoprotein receptor (LDLR) was a potential target of porcine miR-20a (Ding et al., 2019). However, the potential role of miR-20a-5p in human hepatic lipid deposition remains unclear. In the present study, we confirmed that miR-20a-5p is involved in lipid metabolism in hepatocytes. Notably, inhibition of miR-20a-5p significantly aggravated lipid accumulation in hepatocytes, while overexpression of miR-20a-5p reduced lipid accumulation. On account of bioinformatics analysis and luciferase reporter assay, we confirmed that CD36 was the target gene of miR-20a-5p, which can directly bind to the 3′ UTR of CD36, thereby inhibiting the translation of CD36.

CD36 plays an important role in the pathogenesis of chronic liver disease. Early studies focused more on the role of CD36 in atherosclerosis. However, subsequent studies have shown that CD36 is also associated with hepatic simple steatosis (Greco et al., 2008). As a fatty acid translocase, CD36 also acts as a regulator of inflammation and fatty acid oxidation and plays an important role in maintaining intracellular fatty acid homeostasis (Samovski et al., 2015). It was also confirmed in our study that co-transfection of siCD36 and the miR-20a-5p inhibitor effectively reversed the increase of lipid and especially TG level caused by the miR-20a-5p inhibitor under FFA stimulation. A recent study points out that the palmitoylation of CD36 plays a key role in the pathogenesis of NASH by alternating the distribution of CD36 on cell membranes, rather than its expression, which provided a novel functional mode of CD36 (Zhao et al., 2018a). Otherwise, CD36 is also considered to be a negative regulator of autophagy and decrease the expression of CD36 which can correct autophagy defects by activating the AMPK-dependent pathway, thereby inducing lipophagy and reducing lipid overaccumulation (Li et al., 2019). In several mouse strains, CD36 was also identified as the most correlated gene with hepatic TG accumulation and fatty liver (Hui et al., 2015). Lots of studies have also confirmed that CD36 plays an important role in fatty acid uptake and TG accumulation (Hoosdally et al., 2009; Knosgaard et al., 2016). Hepatic fatty acid uptake is mainly through slc27a fatty acid transport proteins (FATP) and the scavenger receptor CD36 (FAT, fatty acid translocase) (Wilson et al., 2016). FATP2 and FATP5 are major FATPs in the liver. However, as shown in Supplementary Figure 3, the mRNA and protein levels of FATP2 and FATP5 showed no significant differences in *vivo* and in *vitro* (Supplementary Figure 3). Only the expression of CD36 was significantly increased in liver tissues of mice fed with high-fat diet.

However, there is an unignorable limitation in this study: we only collected 27 subjects for the detection of plasma miR-20a-5p expression, which led to relatively small sample size.

In summary, this study revealed that the downregulation of miR-20a-5p may lead to the abnormal expression of CD36 in lipid accumulation in NAFLD. In addition, we provide evidence that miR-20a-5p can regulate lipid metabolism in hepatocytes by targeting CD36. These results suggested a novel pathogenesis of NAFLD and may develop potential therapeutic strategies for metabolic diseases.

## Data Availability Statement

The raw data supporting the conclusions of this article will be made available by the authors, without undue reservation, to any qualified researcher.

## Ethics Statement

The studies involving human participants were reviewed and approved by the Ethics Committee of the Capital Medical University, Beijing China. The patients/participants provided their written informed consent to participate in this study. The animal study was reviewed and approved by the Ethics Committee of the Capital Medical University, Beijing China.

## Author Contributions

XW and DZ designed the experiments. YM were responsible for sample collection and database management. XW performed all experiments and data analysis. XW and L-YY wrote the manuscript. DZ supervised the experiments and revised the manuscript. All authors contributed to the article and approved the submitted version.

## Conflict of Interest

The authors declare that the research was conducted in the absence of any commercial or financial relationships that could be construed as a potential conflict of interest.

## Supplementary Material

Supplementary Figure 1 The expression level of CD36 is inversely correlated with miR-20a-5p. **(A)** After 0, 24, 48, and 72 h FFA-treatment, the relative expression level of miR-20a-5p was measured by RT-PCR. **(B)** After 0, 24, 48, and 72 h FFA-treatment, the protein level of CD36 in HepG2 cells was measured by western blot. Data were mean ± SEM. ^∗^*P* < 0.05, ^∗∗^*P* < 0.01. BSA, bovine serum albumin; FFA, free fatty acid; RT-PCR: real-time polymerase chain reaction.

Supplementary Figure 2 The expression level of CD36 is inhibited by miR-20a-5p. The protein level of CD36 in HepG2 cells was measured by western blot after transfection of miR-20a-5p mimic or inhibitor. miR-NC, miR-negative control.

Supplementary Figure 3 The expression level of FATP2, FATP5 *in vivo* and *in vitro*. The mRNA and protein levels of FATP2 and FATP5 in mice **(A,B)** and FFA-treatment HepG2 cells **(C,D)** were measured.
