# Supplementary figures and images for: MicroRNA-20a-5p Ameliorates Non-alcoholic Fatty Liver Disease via Inhibiting the Expression of CD36
Source: Front Cell Dev Biol. 2020 Dec 3;8:596329. doi: 10.3389/fcell.2020.596329 (PMC7744458; doi:10.3389/fcell.2020.596329)

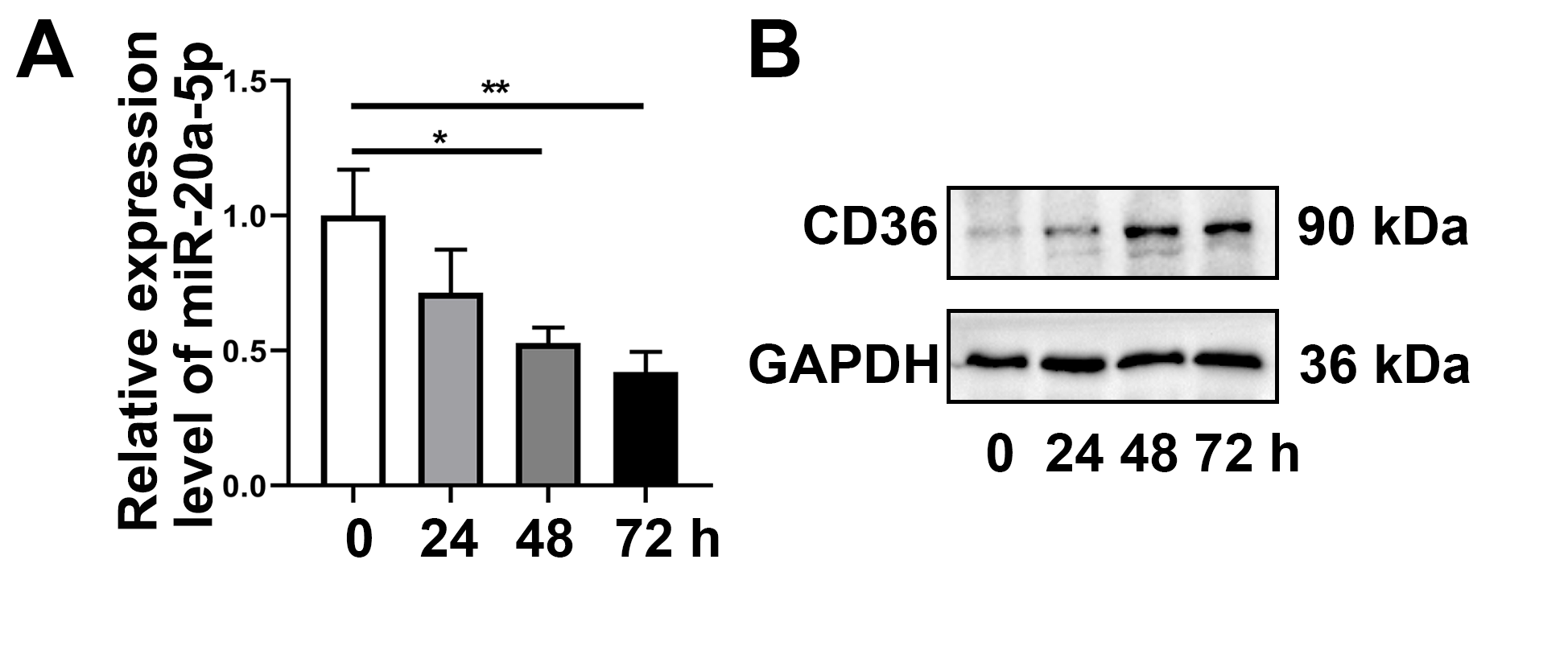

Supplement: Supplementary Figure 1 — The expression level of CD36 is inversely correlated with miR-20a-5p. (A) After 0, 24, 48, and 72 h FFA-treatment, the relative expression level of miR-20a-5p was measured by RT-PCR. (B) After 0, 24, 48, and 72 h FFA-treatment, the protein level of CD36 in HepG2 cells was measured by western blot. Data were mean ± SEM. ∗P < 0.05, ∗∗P < 0.01. BSA, bovine serum albumin; FFA, free fatty acid; RT-PCR: real-time polymerase chain reaction. [file Image_1.TIF]

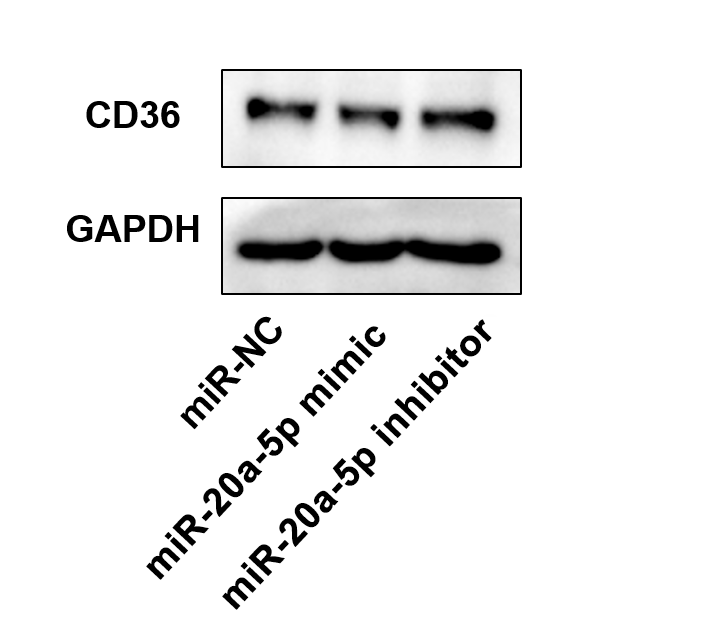

Supplement: Supplementary Figure 2 — The expression level of CD36 is inhibited by miR-20a-5p. The protein level of CD36 in HepG2 cells was measured by western blot after transfection of miR-20a-5p mimic or inhibitor. miR-NC, miR-negative control. [file Image_2.tif]

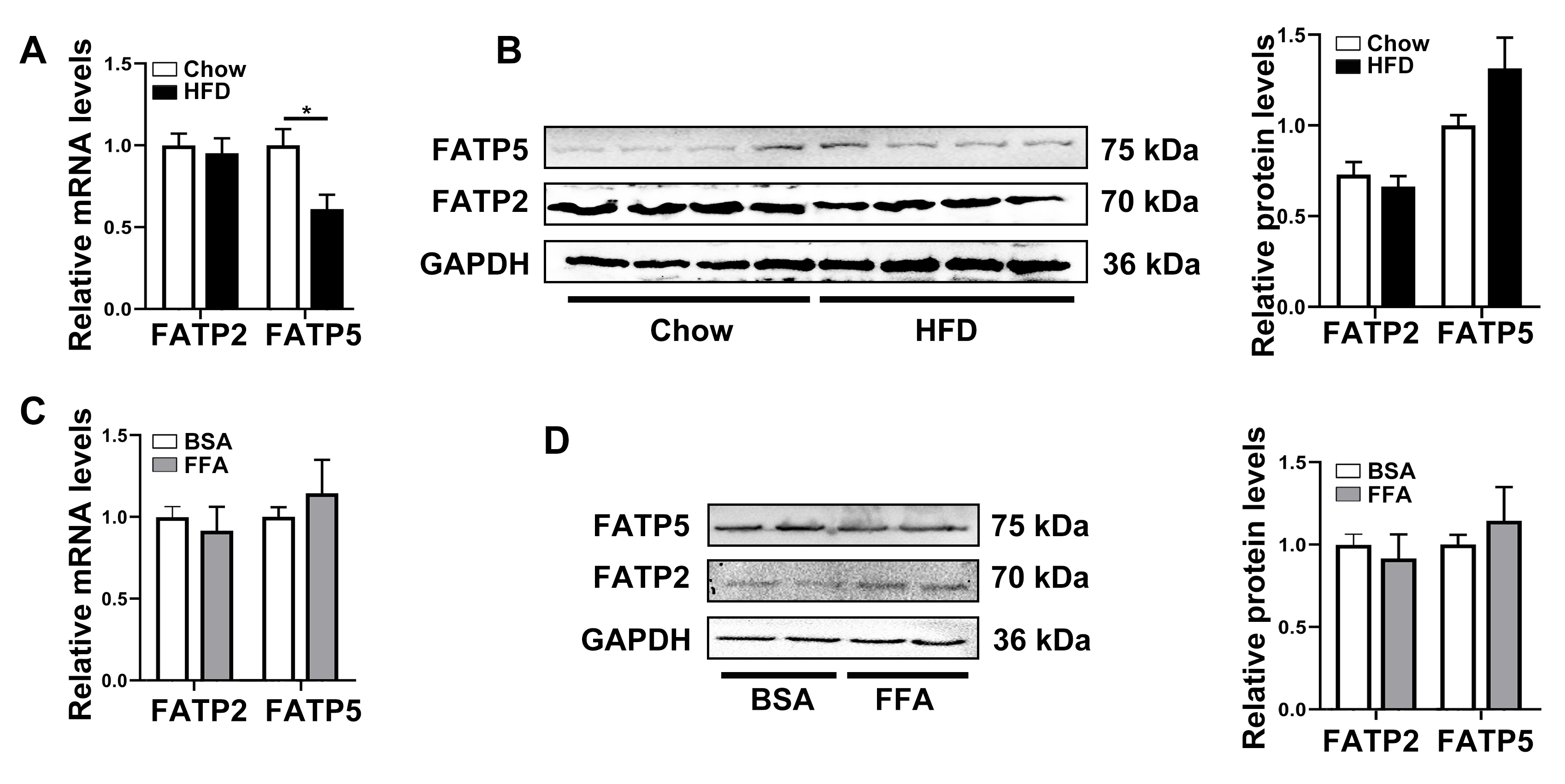

Supplement: Supplementary Figure 3 — The expression level of FATP2, FATP5 in vivo and in vitro. The mRNA and protein levels of FATP2 and FATP5 in mice (A,B) and FFA-treatment HepG2 cells (C,D) were measured. [file Image_3.TIF]
